# Comparative analysis of selective fungal culture media and incubation conditions for *Aspergillus fumigatus* in cystic fibrosis sputum

**DOI:** 10.1128/spectrum.00181-26

**Published:** 2026-05-29

**Authors:** Gina Hong, Elisa M. Vesely, Warda Memon, Joanna Walsh, Jesse Y. Hsu, Anna L. O'Dea, Rebecca H. Dezube, Christopher H. Goss, Jane E. Gross, David P. Nichols, Robert A. Cramer, Sean X. Zhang

**Affiliations:** 1 Department of Medicine, University of Pennsylvania Perelman School of Medicine 14640 , Philadelphia, Pennsylvania, USA; 2 Department of Microbiology and Immunology, Geisel School of Medicine at Dartmouth 12285 , Hanover, New Hampshire, USA; 3 Department of Pathology, Johns Hopkins University School of Medicine 1500 , Baltimore, Maryland, USA; 4 Department of Biostatistics, Epidemiology and Informatics, Perelman School of Medicine, University of Pennsylvania 6572, Philadelphia, Pennsylvania, USA; 5 Center for Clinical Epidemiology and Biostatistics, Perelman School of Medicine, University of Pennsylvania 728235 , Philadelphia, Pennsylvania, USA; 6 Department of Medicine, Johns Hopkins University School of Medicine 1500 , Baltimore, Maryland, USA; 7 Department of Medicine, University of Washington 7284, Seattle, Washington, USA; 8 Departments of Pediatrics and Medicine, National Jewish Health 2930, Denver, Colorado, USA; 9 Departments of Pediatrics and Medicine, University of North Carolina 2331, Chapel Hill, North Carolina, USA; 10 Cystic Fibrosis Foundation 71458, Bethesda, Maryland, USA; Universidade de Sao Paulo Campus de Ribeirao Preto, Sao Paulo, Brazil

**Keywords:** fungal culture, *Aspergillus fumigatus*, cystic fibrosis

## Abstract

*Aspergillus fumigatus* detection in respiratory samples of people with cystic fibrosis (CF) is important for the diagnosis of *Aspergillus*-related lung disease. Yet, fungal culture approaches are not standardized for clinical CF respiratory samples. We analyzed CF sputum to determine culture conditions that may improve the detection of *Aspergillus fumigatus*. A total of 281 remotely collected sputum samples from 74 adults with CF were analyzed in two laboratories, Johns Hopkins and Dartmouth. *Aspergillus fumigatus* was found in 121 (74%) and 84 (71%) samples in Johns Hopkins and Dartmouth, respectively. In the Johns Hopkins laboratory, we observed a decreased odds for *Aspergillus fumigatus* growth when using Czapek-Dox agar (adjusted OR [aOR] 0.78, 95% CI 0.68–0.89, *P* < 0.01) compared to inhibitory mold agar. An incubation temperature of 30°C compared to 37°C was associated with decreased *Aspergillus fumigatus* positivity in 21%O_2_ (aOR 0.71, 95% CI 0.58–0.88, *P* < 0.01). We also observed 82% increased odds for *Aspergillus fumigatus* isolation while incubating in 37°C 1% oxygen (O_2_) compared to 37°C 21% O_2_5% carbon dioxide (CO_2_) (aOR 1.82, 95% CI 1.41–2.36, *P* < 0.01). Additional work in the Dartmouth laboratory demonstrated Sabouraud agar media was associated with decreased odds for *Aspergillus fumigatus* positive culture compared to inhibitory mold agar (aOR 0.77, 95% CI 0.63–0.93, *P* < 0.01). Our data suggest that use of inhibitory mold agar and 37°C and hypoxic incubation conditions may increase the odds to detect *Aspergillus fumigatus* in remotely collected CF sputa. Validation studies are needed to determine if these culture conditions improve *A. fumigatus* detection in CF.

**IMPORTANCE:**

*Aspergillus fumigatus* is the most common filamentous fungus that affects the lungs of people with cystic fibrosis (CF), and the detection is critical to determine whether treatment is required. We examined how fungal culture media, sputum processing, and incubation conditions may impact the recovery of *Aspergillus fumigatus* in CF sputum. Our study found that semi-selective fungal culture media, such as inhibitory mold agar and incubation in 37°C and low oxygen, were associated with *Aspergillus fumigatus* growth in remotely collected CF sputum. Validation of our findings has the potential to impact the approach of clinical fungal culture to detect *Aspergillus fumigatus* in the CF population.

## INTRODUCTION

*Aspergillus fumigatus* is the most common filamentous fungus recovered in the cystic fibrosis (CF) lung (1). *A. fumigatus* and related species affect people with CF in various disease states: *Aspergillus* colonization, bronchitis, allergic bronchopulmonary aspergillosis (ABPA), aspergilloma, and invasive aspergillosis; the latter specifically in organ transplant recipients. Fungal culture is poorly sensitive, yet it remains the clinical gold standard to diagnose fungal respiratory infections in people with CF.

Previous efforts to inform a standardized approach for fungal culture evaluation of CF sputa have been conducted (2–5). Yet, variable laboratory methods continue to influence fungal detection and may impact diagnosis and decisions to implement antifungal therapies. Recent guidelines highlight that most clinical laboratories detect fungi using bacterial culture media, and fungal cultures are often incubated in 30°C (6). However, the biological characteristics of *Aspergillus* spp. and the unique CF airway environment suggest this approach may not be ideal for *Aspergillus* recovery. *Aspergillus fumigatus* thrives at 37°C, which induces rapid germination and hyphal growth of most *Aspergillus* spp.(7–9). Currently, the CF UK trust recommends Sabouraud medium and incubation temperatures of 35–37°C for fungal evaluation of CF respiratory samples (https://www.cysticfibrosis.org.uk/sites/default/files/2023-01/CF%20Lab%20Standards%20FINAL.pdf). Inhibitory mold agar also performs well in the detection of pathogenic molds in CF sputum (2, 6). Given the mucopurulent character of CF sputum, homogenization using mucolytic agents, such as a dithiothreitol (DTT), or sonication to chemically and physically disrupt the sample has been thought to potentially improve recovery of CF pathogens (4). Yet, these processing methods when handling CF sputa have not been implemented in many clinical laboratories. Finally, higher sputum volumes are reported to improve *Aspergillus* detection in CF and non-CF hosts (5, 10). Taken together, there are significant culture variables that likely impact the ability to recover *Aspergillus* spp. from airway samples of people with CF.

As *A. fumigatus* can often persist in the CF airways, the mechanisms in which *A. fumigatus* can adapt in the host environment may inform the ideal culture conditions for detection. *A. fumigatus* exhibits the ability to adapt to low tissue oxygen levels (hypoxic environments) through several mechanisms, many of which are regulated by the fungal sterol regulatory element-binding protein transcription factor, SrbA (11). Hypoxic fitness, as determined by fungal growth, of *A. fumigatus* isolates correlates with increased virulence in non-CF animal models (12). Also, *A. fumigatus* growth, development, and virulence rely significantly on nitrogen metabolism (13, 14). Therefore, we hypothesized that low oxygen incubation conditions and nitrogen rich culture media may facilitate isolation of pathogenic *A. fumigatus* strains.

Remote sampling in CF has increased, likely influenced by the COVID-19 pandemic and rise of telemedicine care delivery in CF care centers in the United States, and is considered favorable by people with CF (15, 16). Current guidance for fungal detection recommends storage of samples at room temperature for up to 24 h if processing and culture are delayed (6). We sought to test the sputum processing steps and culture conditions associated with *A. fumigatus* detection by leveraging a clinical study of remotely collected CF sputum. Fungal detection in people with CF is critical in order to determine when and how individuals should be treated for fungal mediated disease and for monitoring the efficacy of initiated antifungal therapies.

## MATERIALS AND METHODS

We conducted a prospective study of adults with CF from October 2021 to July 2023 as previously described (16). We enrolled adults with CF (18 years or older) without a history of solid organ transplantation who received their CF clinical care from one of the following CF care centers: Johns Hopkins Hospital (Baltimore, Maryland, USA), National Jewish Health (Denver, Colorado, USA), University of Pennsylvania (Philadelphia, Pennsylvania, USA), or University of Washington (Seattle, Washington, USA). Participants self-expectorated sputum once a week for up to four separate occasions during the study and independently shipped the samples in ambient temperature overnight priority shipping via FedEx directly to one of the two laboratories. Alternating samples were received by the Johns Hopkins laboratory and Dartmouth laboratory to test the conditions uniquely equipped by each laboratory. Each participant mailed the first and third sputum collections to the Dartmouth laboratory and the second and fourth collections directly to Johns Hopkins laboratory. Each sputum sample received by the laboratory underwent quality control checks with an assessment of sputum texture and sputum volume measured in milliliters. Sputum texture was assessed by an internally developed qualitative scale and used across the two laboratories. Sputum was grossly characterized as non-mucous (liquidy), semi-mucous (liquidy with partial solid), or mucous (purulent and solid). Our *a priori* study-specific fungal culture protocol aimed to test the hypotheses that culture media, mucolysis with DTT, ultrasonication, a hypoxic incubation environment, with or without 5% CO_2_, and incubation temperature (30°C vs 37°C) affect *A. fumigatus* growth and culture detection in sputum samples of people with CF. Based on total sputum volume, the sputum sample was equally aliquoted, plated on fungal culture media (inhibitory mold agar, Sabouraud agar with gentamicin, and Czapek-Dox agar, a nitrogen-rich culture media), and incubated for up to 10 days. In the Johns Hopkins laboratory (primary lab), we analyzed unprocessed sputum (neat sputum) and sputum undergoing homogenization with DTT or ultrasonication. Then, we incubated plates in the following conditions, 30°C 21% O_2_, 37°C 21% O_2_, 37°C 1% O_2_, 37°C 21% O_2_ 5% CO_2_, and 37°C 1% O_2_ 5% CO_2_. In the Dartmouth laboratory, similar conditions were tested except for ultrasonication, 30°C 21% O_2_, 37°C 21% O_2_, and 37°C 1% O_2_ due to differences in laboratory equipment.

Total sputum volume influenced the number of conditions to be tested in the two laboratories. Johns Hopkins aimed to plate a minimum sputum volume of 10 μL per culture plate. If there was sufficient sputum volume defined as greater than or equal to 0.5 mL of sputum, all conditions (a total of 45 culture plates) were tested. However, if sputum volume was less than 0.5 mL in the Hopkins laboratory, some conditions were unable to be tested. The Dartmouth laboratory tested fewer independent conditions of interest and the protocol split sputum samples into a minimum volume of 100 μL per plate. For the Dartmouth laboratory, if total sputum volume was less than 1.2 mL, the homogenized and unprocessed aliquots were plated on Czapek Dox agar and incubated in 37°C 21% O_2_ 5% CO_2_ or 37°C 1% O_2_ 5% CO_2._ To account for sputum volume in each experiment, we incorporated the volume of sputum inoculated on each plate (sputum volume plated) in our statistical models.

The detection of *A. fumigatus* growth was our primary outcome of interest. If isolated, semi-quantitative *A. fumigatus* growth on each Petri plate was measured (very light, light, medium, or heavy). We identified *A. fumigatus* and other fungal species primarily by macroscopic and microscopic morphology. In the Johns Hopkins laboratory, matrix-assisted laser desorption ionization-time of flight (MALDI-TOF) was also conducted for fungal identification for all fungal isolates. If *A. fumigatus* was identified, *in vitro* antifungal susceptibility testing using Clinical and Laboratory Standards Institute (CLSI) standards was conducted for voriconazole from at least one isolate from each participant. The interpretation to determine voriconazole as susceptible, intermediate, and resistant was based on CLSI clinical breakpoints (17).

### Statistical analysis

Summary statistics were calculated at the subject and sputum collection level. Medians and IQRs are reported for continuous variables, and frequencies and percentages are reported for categorical variables. Due to the protocol differences in the two laboratory settings, we report our results stratified by laboratory. Johns Hopkins was considered the primary analysis since all culture media and incubation conditions were tested. For each laboratory, a multivariable logistic GEE model with an exchangeable correlation structure was fit to examine the associations between testing conditions (culture media, incubation condition, sputum processing methods, and sputum volume plated) and *A. fumigatus* positivity by plate, accounting for repeated measures by subject. We also utilized sensitivity analyses restricting samples with adequate sputum volume to test all the variables of interest (culture media, incubation condition, and sputum processing) in each laboratory. For the Johns Hopkins laboratory, we examined how sputum texture modifies the relationship between sputum processing and *A. fumigatus* positivity by including the sputum texture and its interaction with sputum processing as additional covariates in the model.

## RESULTS

We enrolled 76 adults with CF in the study. Detailed subject characteristics of all 76 enrolled participants and methods for sputum collection are previously described (16). Two individuals were unable to provide any sputum samples due to inability to expectorate sputum. Individual characteristics of the 74 subjects who provided sputum samples are described in Table 1. Median age was 36.0 [29.0, 46.0] years, median forced expiratory volume in 1 s (FEV_1_), percent predicted was 72.0 [53.3, 85.5], and 64 (86.4%) reported taking CFTR modulator therapy.

**TABLE 1:** Cohort characteristics^*b*^

	Overall (*N* = 74)
Cystic Fibrosis Center and Region	
University of Pennsylvania, Pennsylvania	25 (33.8%)
Johns Hopkins University, Maryland	24 (32.4%)
National Jewish Health, Colorado	13 (17.6%)
University of Washington, Washington	12 (16.2%)
Age, median [IQR], years	36.0 [29.0, 46.0]
Female sex	45 (60.8%)
White race	67 (90.5%)
Hispanic/LatinX ethnicity	3 (4.1%)
Pancreatic insufficiency	63 (85.1%)
FEV_1_ percent predicted, median [IQR], %	72.0 [53.3, 85.5]
CFTR modulators	64 (86.4%)
Trikafta (Elexacaftor/Tezacaftor/Ivacaftor)	59 (79.7%)
Symdeko (Tezacaftor/Ivacaftor)	3 (4.1%)
Kalydeco (Ivacaftor)	2 (2.7%)
Antifungals^***a***^	3 (4.0%)
*Aspergillus* history	44 (59.5%)
*Pseudomonas aeruginosa* history	47 (63.5%)
Methicillin-sensitive *Staphylococcus aureus* history	39 (52.7%)
MRSA history	11 (14.9%)

^*a*^ Voriconazole 1 subject, Posaconazole 1 subject, and Isavuconazole in 1 subject.

^*b*^ FEV_1_ = forced expiratory volume in one second, IQR= interquartile range, CFTR= cystic fibrosis transmembrane conductance regulator, MRSA= methicillin-resistant *Staphylococcus aureus.*

The Dartmouth laboratory was unable to receive samples from 11 participants from the periods between April 2022 to May 2022 and July 2022 to September 2022 (see Supplement). During these time periods, the 11 participants sent all 4 sputum samples to the Johns Hopkins laboratory for analysis. A total of 284 sputum samples were received by the laboratories and 281 were deemed suitable for analysis. Two samples with undetectable volume and one sample that was dark brown in color after being received 8 days after collection were omitted from the culture analysis. Of the 281 samples received and analyzed by the laboratories, 235 (83.6%) arrived within 1 day of collection. Median [IQR] total sputum volume was 2.0 [1.0, 4.0] mL. We observed 123 (43.8%) mucous, 78 (27.8%) semi-mucous, and 80 (28.5%) non-mucous sputum samples. *A. fumigatus* was detected in 205 (73%) samples.

### Experimental conditions associated with *A. fumigatus* detection in each laboratory

#### Johns Hopkins laboratory

A total of 163 samples from 73 participants were received. A total of 6,894 plates were analyzed given the multiple variables tested. Table S1 summarizes the number of plates tested for each culture medium, incubation condition, and sputum processing variable.

##### A. fumigatus

**Fig 1:**
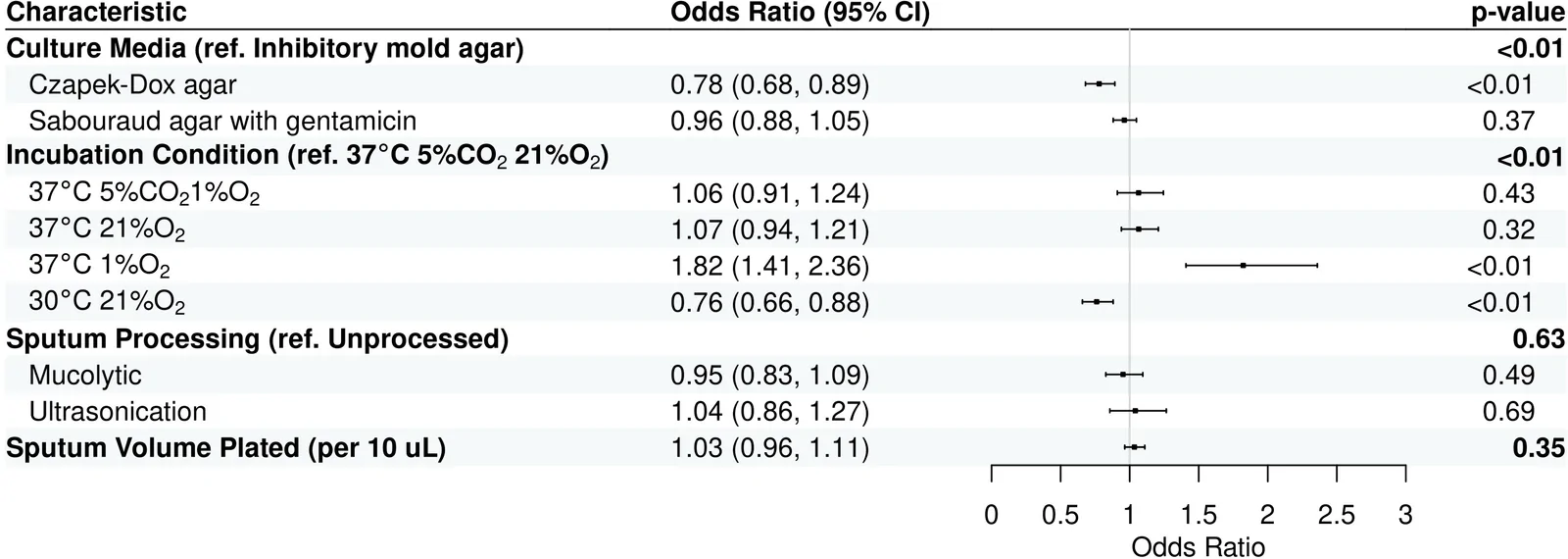
Fungal culture media, 30°C, and 1% O_2_ with ambient CO_2_ impacts *Aspergillus fumigatus* detection in remote sputum samples of adults with cystic fibrosis, accounting for sputum volume plated, in the Johns Hopkins laboratory.

*A. fumigatus* was detected in 62 (84.9%) patients and 121 (74.2%) samples. *A. fumigatus* was found in at least 50% of the plates cultured in 11 individuals. We found that culture media and incubation condition were significantly associated with *A. fumigatus* positivity after accounting for sputum volume plated (*P* < 0.01 and *P* < 0.01, respectively, Fig. 1). Czapek Dox agar was associated with 0.78 lower odds for *A. fumigatus* positive culture compared to inhibitory mold agar as the reference (aOR 0.78, 95% CI 0.68–0.89, *P* < 0.01). For incubation conditions, the odds of *A. fumigatus* growth when incubated at 30°C 21% O_2_ were 0.76 times the odds when incubated at 37°C 21% O_2_ 5% CO_2_ (aOR 0.76, 95% CI 0.66–0.88, *P* < 0.01). Compared to 37°C 21% O_2_ (as the reference), the odds for *A. fumigatus* growth in 30°C 21% O_2_ was also reduced (aOR 0.71, 95% CI 0.58–0.88, *P* < 0.01). Conversely, the odds of *A. fumigatus* growth when incubated at 37°C 1% O_2_ were 1.82 times the odds when incubated at 37°C 21% O_2_ 5% CO_2_ (aOR 1.82, 95% CI 1.41–2.36, *P* < 0.01). However, we did not observe a significant difference between incubation conditions of 37°C 1% O_2_ 5% CO_2_ and 37°C 21% O_2_ 5% CO_2_ in their association with *A. fumigatus* growth (aOR 1.06, 95% CI 0.91–1.24, *P* = 0.43). Sputum processing methods were not significantly associated with *A. fumigatus* positivity (aOR 0.95, 95% CI 0.83–1.09, *P* = 0.49 and aOR 1.04, 95% CI 0.86–1.27, *P* = 0.69 for mucolysis with DTT and ultrasonication, respectively). Also, we did not observe effect modification of sputum texture and sputum processing (*P* for interaction = 0.25). Similar relationships were observed in our sensitivity analysis including only the 137 sputum samples that had sufficient total sputum volume to test all 45 combinations of the variables of interest (Table S2). These data suggest that plating on inhibitory mold agar and incubating at 37°C and 1% O_2_ (with ambient CO_2_) may facilitate *A. fumigatus* growth when present in CF sputum.

##### Other fungal prevalence

In addition to *A. fumigatus*, non-*fumigatus Aspergillus* species and other fungal species were detected in remote sputum samples (Fig. S1). The most prevalent filamentous fungi among participants were *Penicillium* species (34.3%), *Exophiala* species (19.2%), *Aspergillus niger* (13.7%), and *Basidiomycetes* (12.3%). Rare species were collapsed into an “Other” category and are outlined in the supplement.

### Dartmouth laboratory

A total of 118 sputum samples from 62 participants were received. A total of 876 plates were analyzed as certain conditions tested at Johns Hopkins were not tested at this site due to equipment limitations. Table S3 summarizes the number of plates tested for each variable.

#### A. fumigatus

**Fig 2:**
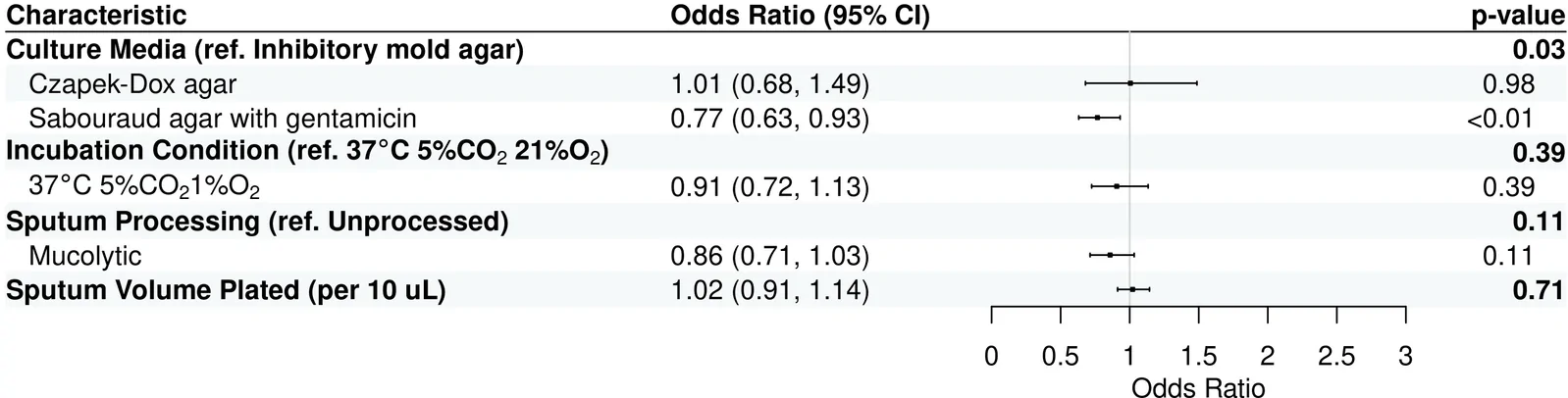
Fungal culture media impacts *Aspergillus fumigatus* detection in remote sputum samples in adults with CF, accounting for sputum volume plated, in the Dartmouth laboratory.

*A. fumigatus* was detected in 50 participants (80.7%) and 84 samples (71.2%). Twenty-eight (45.2%) participants had *A. fumigatus* detected in at least 50% of plates. We observed that culture media was associated with *A. fumigatus* detection (*P* = 0.03, Fig. 2). Czapek-Dox agar was not associated with *A. fumigatus* positive culture compared to inhibitory mold agar as the reference (aOR 1.01, 95% CI 0.68–1.49, *P* = 0.98). However, Sabouraud dextrose agar with gentamicin was significantly associated with 23% lower odds of *A. fumigatus* positivity compared to inhibitory mold agar (aOR 0.77, 95% CI 0.63–0.93 *P* < 0.01) when accounting for incubation condition, sputum processing, and sputum volume plated. Similar to Johns Hopkins laboratory, we did not observe a relationship between 1% O_2_ and 5% CO_2_ incubation conditions and *A. fumigatus* detection compared to 21% O_2_ and 5% CO_2_ at 37°C (aOR 0.91, 95% CI 0.72–1.13, *P* = 0.39). Mucolysis was not associated with *A. fumigatus* positivity (aOR 0.86, 95% CI 0.71–1.03, *P* = 0.11). Sensitivity analyses were conducted including only the 57 samples that had sufficient sputum volume for testing all 12 available experimental conditions at Dartmouth laboratory. We found a similar relationship between Sabouraud dextrose agar and decreased *A. fumigatus* positivity (Table S4).

#### Other fungal prevalence

Dartmouth identified additional fungal isolates in participants, including *Aspergillus nidulans* in 4 (6.5%) patients, *Paecilomyces* species in 4 (6.5%) patients, and *Aspergillus terreus* in 2 (3.2%) patients. Additional fungi found in samples are described in Fig. S2.

### Antifungal susceptibility testing

#### Johns Hopkins laboratory

A total of 65 *A. fumigatus* isolates from 62 participants underwent voriconazole susceptibility testing. We found 54 (83.1%) susceptible, 10 (15.4%) intermediate, and 1 (1.5%) resistant isolate to voriconazole. Three participants had antifungal susceptibility testing done on two different isolates. Of the three participants, two participants had unique isolates exhibiting different antifungal susceptibility patterns; one *A. fumigatus* isolate was susceptible and the other intermediate to voriconazole.

#### Dartmouth laboratory

A total of 38 *A. fumigatus* isolates from 38 distinct participants underwent voriconazole susceptibility testing. We found 36 (94.7%) *A*. *fumigatus* isolates susceptible to voriconazole and 2 (5.3%) intermediate to voriconazole. We did not observe azole resistance in the Dartmouth laboratory.

## DISCUSSION

We leveraged a prospective observational study collecting remote sputum samples from adults with CF to investigate laboratory conditions that may optimize the detection of *A. fumigatus* based on studies associating specific environmental conditions with *Aspergillus* pathogenicity and virulence. Although microbiologic cultures of remote respiratory samples have yet to be clinically validated, there has been a rising interest in the utilization of remotely collected sputum samples in the CF population due to the marked decreases in spontaneously expectorated sputum samples in clinical settings. As existing CF microbiology guidelines suggest delays in CF sputum processing for fungal detection can be up to 24 h at room temperature, we hypothesized that *A. fumigatus* when present would exhibit stability in CF sputum for overnight transport from the patient’s home directly to the laboratory. *A. fumigatus* detection was higher than expected in our cohort consistently in both laboratories, as compared to recent data supporting a potential decrease in *A. fumigatus* prevalence in people with CF on elexacaftor/tezacaftor/ivacaftor (18, 19). Although it is possible that environmental recovery of *A. fumigatus* in these delayed processed sputa occurred, an alternative explanation is that processing and incubation conditions that were tested may have enhanced the overall recovery of *A. fumigatus* in CF sputum. While not a direct comparison, we did ship sterile and *A. fumigatus* spiked medium samples from Dartmouth to Hopkins laboratory under similar conditions as those utilized by study participants. Control (non-*Aspergillus* spiked samples) did not culture *Aspergillus* despite coming from an environment at Dartmouth with heavy *Aspergillus* laboratory usage. Importantly, our study was not designed to fully address the validity of *A. fumigatus* culture results in remotely collected CF sputum, given the absence of paired clinic-collected samples.

Existing data support that high sputum volumes and homogenization should be considered for fungus culture and optimal *A. fumigatus* recovery (4, 10, 20). Yet, sputum processing (mucolysis or ultrasonication) was not observed to be associated with *A. fumigatus* detection. Furthermore, we did not observe that mucous texture of sputum affected the relationship between mucolysis or ultrasonication and *A. fumigatus* positivity. The sputum in our contemporary CF cohort may exhibit reduced viscoelasticity given the effects of elexacaftor/tezacaftor/ivacaftor compared to historical CF cohorts without CFTR modulators, which may explain our findings (21). The relationship between sputum volume and *A. fumigatus* positivity cannot be clearly defined in our study due to the study’s design to split the total sputum volume into approximately equal aliquots to compare the plates and conditions.

Currently, many clinical laboratories worldwide implement Sabouraud agar with gentamicin and inhibitory mold agar for fungal culture for clinical samples, including CF sputum and bronchoalveolar samples. Based on elevated levels of nitrate being present in many CF sputum samples and *A. fumigatus’s* ability to use nitrate as a sole nitrogen source, we tested the role of nitrate containing Czapek-Dox agar in our study. However, Czapek-Dox was found to be negatively associated with *A. fumigatus* detection compared to inhibitory mold agar in the Johns Hopkins laboratory. Inhibitory mold agar contains multiple nitrogen sources including casein and meat-based peptone in addition to other nitrogen sources. One potential explanation for the poor recovery on Czapek-Dox agar media is that fungal isolates over time develop alterations in their ability to utilize non-preferred nitrogen sources such as nitrate through nitrogen catabolite repression regulation. Additionally, given the high likelihood of bacterial co-infection and the high prevalence of *Pseudomonas aeruginosa* in the cohort, it is possible that the absence of antibiotic enrichment in the Czapek-Dox agar media may have impacted our findings. Though *Pseudomonas aeruginosa* and *A. fumigatus* co-infection is common in people with CF, pathogen interactions exist which inhibit the *in vitro* growth of *Pseudomonas* and *A. fumigatus* on co-culture (7, 22–26). Based on the additional data from the Dartmouth lab, Sabouraud agar with gentamicin was associated with lower odds of *A. fumigatus* positivity compared to inhibitory mold agar. Altogether, inhibitory mold agar may be the ideal culture media to consider for detecting *A. fumigatus* when accounting for all other processing, incubation conditions, and sputum volume. However, these data require further validation.

*A. fumigatus* has robust growth mechanisms in hypoxic microenvironments found in CF lung environments that can vary across isolates (27). Regulatory genes encoding *A. fumigatus* transcription factors important for hypoxia growth include SrbA, SrbB, AtrR, and CreA, and these genes are subsequently critical for *A. fumigatus* growth in hypoxic environments and associated with virulence (28–30). Morphology of *A. fumigatus* colonies and biofilms in hypoxic conditions are also distinctly different from those in normoxic conditions, and these morphologies are associated with different inflammatory profiles (31). Given the connections between *A. fumigatus* pathobiology and low oxygen conditions commonly found in CF lungs, we examined the impact of culture in low oxygen (hypoxic) conditions. Intriguingly, we observed increased odds of *A. fumigatus* isolation in hypoxic incubation conditions compared to normoxia. However, surprisingly, we observed the significant association with low oxygen environments and *A. fumigatus* growth in ambient CO_2_ conditions (approximately 0.04%), which is significantly lower than 5%. Perhaps consistent with these observations, a longitudinal study of *A. fumigatus* isolates in people with CF revealed a cluster of isolates persisting for at least 5 years possessing reduced growth in normoxic conditions versus hypoxic conditions (32). Taken together, these data lead to the hypothesis that *A. fumigatus* isolates persistent in CF adapt to the low oxygen environment and become more difficult to culture in common laboratory conditions. It is currently unclear why physiological levels of CO_2_ normalize the recovery of *A. fumigatus* across oxygen levels tested in this study (ambient [21%] vs 1% O_2_). It is possible that high CO_2_ levels promote growth of isolates adapted to the CF lung environment regardless of O_2_ levels. Rigorous investigation of hypoxic incubation conditions and the relationship between CO_2_ and O_2_ levels in determining *A. fumigatus* growth is required to further validate and understand these results.

Antifungal resistance is a threat to fungal infections and has been described among CF cohorts at a higher level than the general population (33). Previous exposure to antifungal therapies increases risk for azole resistance, but *de novo* resistance from the environment is also described (34, 35). Voriconazole resistance was rare in our cohort. Yet, minimum inhibitory concentration reflected intermediate susceptibility to voriconazole in 12 *A. fumigatus* isolates. Only three participants reported antifungal use despite high proportion of *A. fumigatus* history (two reports of posaconazole and one receiving isavuconazole). Antifungals are often not prescribed for *A. fumigatus* positive cultures in CF, except in specific clinical scenarios, due to the unclear clinical effectiveness (36, 37). A significant issue in addressing when to use antifungals in people with CF is the lack of definitive approaches to determine transient fungal colonization vs persistent fungal presence in the lungs.

Our study proposes laboratory considerations for fungal culture in CF sputa, including use of inhibitory mold agar enriched with antibiotics and incubation temperature at 37°C, and provokes further questions about the potential role of hypoxic incubation conditions to improve the detection of *A. fumigatus* isolates from the human host. We acknowledge several limitations to our study, which must be taken into account. First, we attempted to standardize the testing of the remote CF sputum across both the Johns Hopkins and Dartmouth laboratories. However, due to equipment (e.g., hypoxia chambers, number of incubators) and access differences among the laboratories, we were unable to test all variables in the experiments across both laboratories. Therefore, we stratified our analysis by laboratory with a focus on the Johns Hopkins results and accounted for additional factors in our analysis (e.g., sputum volume plated). We were unable to directly compare the reproducibility and consistency of our findings across laboratories though we note the overall *A. fumigatus* culture positivity across samples was similar between the two laboratories which is noteworthy given the differences in approach. The remote collection of sputum and shipment to the laboratory resulting in delayed processing may impact our findings. Environmental contamination of the unsupervised sputum collection at home is possible, but low likelihood as evidenced by our controlled pilot experiment shipping media between laboratories. The validity of remotely collected respiratory samples in people with CF has yet to be defined, but recent data suggest minimal bias in delayed processing (38–40). Infection surveillance using sputum collected at home is highly pragmatic in the CF population and examining the validity using remotely collected sputum for *A. fumigatus* detection is the necessary next step. An active multi-center study comparing pathogen detection, including *Aspergillus* species, in remotely collected CF sputa and induced sputum collected in clinic aims to test the accuracy and validity of this sampling method (NCT06950892). A single-center validation study replicating our current protocol is ongoing using clinic-collected sputa in a convenience sample of people with CF to further address the limitations of remotely collected samples.

## SUPPLEMENTAL MATERIAL

Supplemental materials spectrum.00181-26-s0001.docx Supplemental text, Tables S1 to S4, and Figures S1 and S2.

ASM does not own the copyrights to Supplemental Material that may be linked to, or accessed through, an article. The authors have granted ASM a non-exclusive, world-wide license to publish the Supplemental Material files. Please contact the corresponding author directly for reuse.
