# Supplementary material for: Comparative analysis of selective fungal culture media and incubation conditions for Aspergillus fumigatus in cystic fibrosis sputum
Source: Microbiol Spectr. 2026 May 29;14(7):e00181-26. doi: 10.1128/spectrum.00181-26 (PMC13339968; doi:10.1128/spectrum.00181-26)

**Supplemental Text**

**Study design**

During the periods of April 2022 to May 2022 and July 2022 to September 2022, all remote sputum samples were shipped to the Johns Hopkins laboratory due to a temporary Dartmouth laboratory closure due to unrelated laboratory circumstance and personnel changes respectively. As a result, 11 participants sent all four sputum samples to the Johns Hopkins laboratory for analysis and followed the Johns Hopkins laboratory study protocol accordingly.

**Supplemental Tables/Figures**

**Table S1**: Summary statistics for the conditions of interest, stratified by *Aspergillus fumigatus* culture positivity for Johns Hopkins laboratory

| **Experimental variables** | **Negative   (N=5335)** | **Positive   (N=1559)** | **Overall   (N=6894)** |
| --- | --- | --- | --- |
| **Culture Media** |  |  |  |
| **Inhibitory mold agar** | 1768 (33.1%) | 564 (36.2%) | 2332 (33.8%) |
| **Czapek-Dox agar** | 1783 (33.4%) | 448 (28.7%) | 2231 (32.4%) |
| **Sabouraud agar with gentamicin** | 1784 (33.4%) | 547 (35.1%) | 2331 (33.8%) |
| **Incubation Condition** |  |  |  |
| **37°C 5%CO_2_ 21%O_2_** | 1105 (20.7%) | 298 (19.1%) | 1403 (20.4%) |
| **37°C 5%CO_2_ 1%O_2_** | 1064 (19.9%) | 292 (18.7%) | 1356 (19.7%) |
| **37°C 21%O_2_** | 1098 (20.6%) | 311 (19.9%) | 1409 (20.4%) |
| **37°C 1%O_2_** | 920 (17.2%) | 427 (27.4%) | 1347 (19.5%) |
| **30°C 21%O_2_** | 1148 (21.5%) | 231 (14.8%) | 1379 (20.0%) |
| **Sputum Processing** |  |  |  |
| **Unprocessed** | 1806 (33.9%) | 518 (33.2%) | 2324 (33.7%) |
| **Mucolytic** | 1783 (33.4%) | 487 (31.2%) | 2270 (32.9%) |
| **Ultrasonication** | 1746 (32.7%) | 554 (35.5%) | 2300 (33.4%) |
| **Texture** |  |  |  |
| **NM** | 1287 (24.1%) | 251 (16.1%) | 1538 (22.3%) |
| **SM** | 1561 (29.3%) | 691 (44.3%) | 2252 (32.7%) |
| **M** | 2487 (46.6%) | 617 (39.6%) | 3104 (45.0%) |
| **Sputum Volume Plated** |  |  |  |
| **Median [Q1, Q3]** | 60.0 [30.0, 100] | 70.0 [40.0, 100] | 60.0 [30.0, 100] |

**Table S2**: The relationship between culture media, incubation condition, and sputum processing and *Aspergillus fumigatus* positive cultures (adjusting for sputum volume plated) in plates of 137 samples from 67 adults with CF with sufficient sputum volume allowing for all experimental variables to be tested in the Johns Hopkins laboratory.

| **Experimental variable** | **OR (95% CI)** | **p-value** |
| --- | --- | --- |
| **Culture Media** |  | <0.01 |
| **Inhibitory mold agar** |  |  |
| **Czapek-Dox agar** | 0.75 (0.64, 0.87) | <0.01 |
| **Sabouraud agar with gentamicin** | 0.97 (0.88, 1.07) | 0.52 |
| **Incubation Condition** |  | <0.01 |
| **37°C 5%CO_2_ 21%O_2_** |  |  |
| **37°C 5%CO_2_ 1%O_2_** | 1.09 (0.9, 1.31) | 0.37 |
| **37°C 21%O_2_** | 1.13 (0.97, 1.31) | 0.11 |
| **37°C 1%O_2_** | 1.96 (1.45, 2.64) | <0.01 |
| **30°C 21%O_2_** | 0.75 (0.64, 0.89) | <0.01 |
| **Sputum Processing** |  | 0.46 |
| **Unprocessed** |  |  |
| **Mucolytic** | 0.94 (0.8, 1.1) | 0.42 |
| **Ultrasonication** | 1.08 (0.88, 1.34) | 0.46 |
| **Sputum Volume Plated (per 10 uL)** | 1.02 (0.94, 1.11) | 0.61 |

**Figure S1**. Fungal prevalence in sputum among 73 participants reported by Hopkins laboratory. Number at the top of the bar indicated count (n). “Other” category includes rare species, including *Scedosporium apiospermum* (described in Table below).


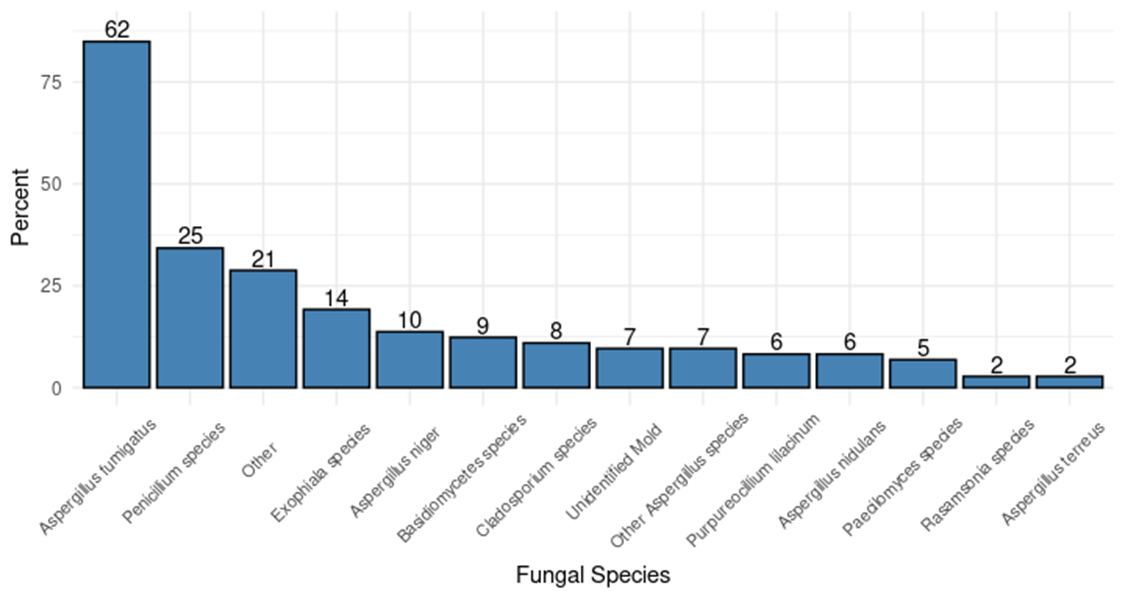


Note: Species identified in the “Other” category in Figure S1 among 21 participants

| **Other fungi** | **N (%)** |
| --- | --- |
| *Alternaria* species | 2 (2.74%) |
| *Curvularia* Species | 2 (2.74%) |
| *Fusarium* species | 2 (2.74%) |
| *Fusarium oxysporum* | 2 (2.74%) |
| *Fusarium solani* species complex | 2 (2.74%) |
| *Candida albicans* | 1 (1.37%) |
| *Chaetomium cristatum* | 1 (1.37%) |
| *Cunninghamella bertholletiae* | 1 (1.37%) |
| *Fusarium fujikuroi* species complex | 1 (1.37%) |
| *Hyphodontia microspora* | 1 (1.37%) |
| *Onchroconis constricta* | 1 (1.37%) |
| *Paraconiothyrium brasiliense* | 1 (1.37%) |
| *Phlebia radiata* | 1 (1.37%) |
| *Scedosporium apiospermum* | 1 (1.37%) |
| *Syncepahastrum monosporum* | 1 (1.37%) |
| *Thermomyces lanuginosus* | 1 (1.37%) |

**Table S3:** Summary statistics for the conditions of interest, stratified by *Aspergillus fumigatus* culture positivity performed in the Dartmouth laboratory

| **Experimental variables** | **Negative   (N=504)** | **Positive   (N=372)** | **Overall   (N=876)** |
| --- | --- | --- | --- |
| **Culture Media** |  |  |  |
| **Inhibitory mold agar** | 147 (29.2%) | 115 (30.9%) | 262 (29.9%) |
| **Czapek-Dox agar** | 193 (38.3%) | 158 (42.5%) | 351 (40.1%) |
| **Sabouraud agar with gentamicin** | 164 (32.5%) | 99 (26.6%) | 263 (30.0%) |
| **Incubation Condition** |  |  |  |
| **37°C 5%CO_2_ 21%O_2_** | 247 (49.0%) | 192 (51.6%) | 439 (50.1%) |
| **37°C 5%CO_2_ 1%O_2_** | 257 (51.0%) | 180 (48.4%) | 437 (49.9%) |
| **Sputum Processing** |  |  |  |
| **Unprocessed** | 303 (60.1%) | 225 (60.5%) | 528 (60.3%) |
| **Mucolytic** | 201 (39.9%) | 147 (39.5%) | 348 (39.7%) |
| **Texture** |  |  |  |
| **NM** | 141 (28.0%) | 97 (26.1%) | 238 (27.2%) |
| **SM** | 134 (26.6%) | 74 (19.9%) | 208 (23.7%) |
| **M** | 229 (45.4%) | 201 (54.0%) | 430 (49.1%) |
| **Sputum Volume Plated** |  |  |  |
| **Median [Q1, Q3]** | 100 [100, 100] | 100 [100, 100] | 100 [100, 100] |

**Table S4**: The relationship between culture media, incubation condition, and sputum processing and *Aspergillus fumigatus* positive cultures (adjusting for sputum volume plated) from 57 samples from 42 adults with CF with sufficient sputum volume allowing for all experimental variables to be tested in the Dartmouth laboratory.

| **Experimental variable** | **OR (95% CI)** | **p-value** |
| --- | --- | --- |
| **Culture Media** |  | 0.03 |
| **Inhibitory mold agar** |  |  |
| **Czapek-Dox agar** | 0.98 (0.61, 1.57) | 0.94 |
| **Sabouraud agar with gentamicin** | 0.78 (0.64, 0.94) | <0.01 |
| **Incubation Condition** |  | 0.17 |
| **37°C 5%CO_2_ 21%O_2_** |  |  |
| **37°C 5%CO_2_ 1%O_2_** | 0.82 (0.63, 1.09) | 0.17 |
| **Sputum Processing** |  | 0.45 |
| **Unprocessed** |  |  |
| **Mucolytic** | 0.94 (0.79, 1.11) | 0.45 |
| **Sputum Volume Plated (per 10 uL)** | 0.91 (0.76, 1.1) | 0.34 |

**Figure S2**. Fungal prevalence in sputum among 62 participants reported by Dartmouth laboratory. Number at the top of the bar indicated count (n).


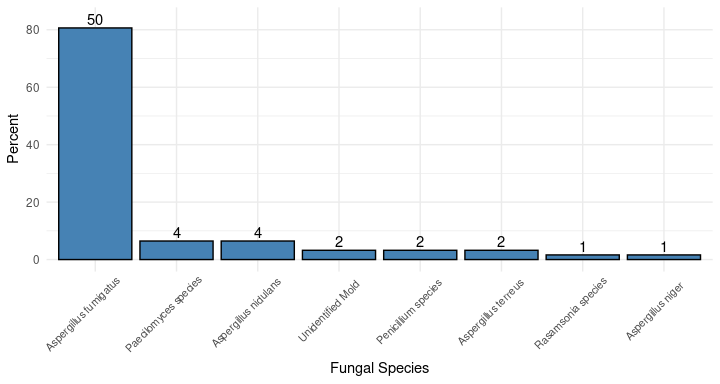

Supplement: Supplemental materials — Supplemental text, Tables S1 to S4, and Figures S1 and S2. [file spectrum.00181-26-s0001.docx]
